# Ferroptosis-related oxidative stress activation in the acute phase of Kawasaki disease

**DOI:** 10.3389/fimmu.2025.1704978

**Published:** 2025-11-13

**Authors:** Xing Xue, Guangan Dai, Yixiang Lin, Lan He, Wei Sheng, Jingwei Sun, Fang Liu

**Affiliations:** 1 Heart Center, Children’s Hospital of Fudan University, Shanghai, China; 2 Pediatrics Department, Bengbu First People’s Hospital, Bengbu, China

**Keywords:** Kawasaki disease, ROS, macrophages, ferroptosis, Liproxstatin-1

## Abstract

**Introduction:**

Kawasaki disease (KD) is an acute systemic vasculitis with unclear etiology and pathogenesis. Emerging evidence suggests a potential role of ferroptosis in various cardiovascular diseases. Therefore, this study aimed to explore and validate the involvement of ferroptosis in the pathogenesis of KD.

**Methods:**

Peripheral blood samples were collected from 10 patients with KD and 10 febrile controls (FC) were collected for RNA sequencing. Differentially expressed genes (DEGs) were identified and subjected to pathway enrichment analysis. Ferroptosis-related DEGs were further validated in patient samples. In vitro, THP-1-derived macrophages (THP-1-Mφs) were stimulated with sera from KD patients to assess intracellular Fe2+ accumulation, lipid reactive oxygen species (lipid ROS) levels, mitochondrial membrane potential, and mitochondrial ultrastructural changes. Liproxstatin-1, a specific ferroptosis inhibitor, was applied to determine whether the ferroptosis-related alterations were reversible.

**Results:**

Transcriptomic analysis revealed significant enrichment of DEGs in the ferroptosis pathway. Validation experiments confirmed a trend toward ferroptosis activation in KD patients. In vitro, THP-1-Mφs treated with KD sera exhibited increased intracellular Fe2+ and lipid ROS levels, impaired mitochondrial membrane potential, and characteristic mitochondrial morphological alterations associated with ferroptosis. Notably, these ferroptosis-related changes were attenuated by Liproxstatin-1 treatment.

**Conclusion:**

Our findings indicate that ferroptosis is activated in KD and may contribute to its pathogenesis. Ferroptosis inhibitor alleviated the associated cellular damage, suggesting that it may represent a potential therapeutic strategy for KD.

## 1 Introduction

Kawasaki disease (KD) is an acute systemic vasculitis that predominantly affects the coronary arteries (1). Intravenous immunoglobulin (IVIG) combined with aspirin is the standard treatment during the acute phase of KD (2–4), effectively reducing inflammation and preventing the development and progression of coronary artery lesions (CALs). However, 8-25% of patients are unresponsive to this therapy, which is a known risk factor for CAL (2–4).

Although the pathogenesis of KD remains unclear, multiple factors have been implicated, including inflammation, oxidative stress and endothelial dysfunction (5, 6). In particular, levels of reactive oxygen species (ROS) are significantly elevated during the acute phase and decreased following IVIG treatment (7). Biomarkers such as elevated urinary 8-iso-prostaglandin F2α have been reported to support the role of oxidative stress as a key mediator of KD-related inflammation (8). Excessive ROS production not only exacerbates oxidative stress but also initiates a self-perpetuating inflammatory cycle that contributes to the progression of vasculitis (9). These findings underscore the potential benefit of targeting oxidative stress as an adjunctive therapeutic strategy for KD, especially in IVIG-resistant patients.

Ferroptosis is a distinct form of regulated cell death driven by iron-dependent lipid peroxidation. It involves multiple metabolic processes, including redox balance, iron homeostasis, mitochondrial function, and amino acid and lipid metabolism (10). This process is often accompanied by the accumulation of mitochondrial ROS, which further amplifies oxidative damage (11). Recent studies have shown the involvement of ferroptosis in various cardiovascular diseases, such as doxorubicin-induced cardiomyopathy (12, 13), ischemia–reperfusion injury (14, 15), myocardial infarction (16, 17), and heart failure (18, 19). In the context of KD, serum ferritin levels, which reflect iron overload, are higher than those in other febrile illnesses (20, 21). Ferritin has also been proposed as a biomarker for predicting IVIG resistance and severe form of KD such as macrophage activation syndrome (MAS) (22–24). But the involvement of ferroptosis in KD pathogenesis has not been thoroughly elucidated.

To investigate the potential role of ferroptosis in the acute phase of KD, we performed RNA sequencing (RNA-seq) and bioinformatics analyses, followed by validation using clinical samples and THP-1-derived macrophage (THP-1-Mφ) models. The study aims to explore whether ferroptosis participate in the pathogenesis of KD.

## 2 Methods

### 2.1 Study population

From December 2023 to December 2024, patients diagnosed with KD (KD group) and patients with other febrile illnesses (fever control group, FC group) were prospectively recruited at the Children’s Hospital of Fudan University in a 1:1 age-matched ratio. A total of 84 patients were enrolled (KD: FC = 42:42). Peripheral blood leukocyte samples from 10 patients per group were randomly selected for RNA-seq, while leukocytes from the remaining patients were used for real-time fluorescence quantitative PCR (qPCR) validation. Serum samples from both the KD and FC groups were collected for stimulation experiments using THP-1-Mφs. In addition, sera from 42 healthy children (healthy control group, HC group) were collected and used as a baseline control.

The diagnosis of KD was based on the 2017 American Heart Association guidelines (2), and all blood samples from the KD group were collected during the acute phase, prior to IVIG and aspirin treatment. FC group were defined as children with a body temperature ≥38°C and without clinical features suggestive of KD. The febrile illnesses were primarily uncomplicated respiratory or gastrointestinal infections. HC group were defined as afebrile healthy children with no evidence of infection at the time of sampling.

This study was approved by the Institutional Review Boards of the Children’s Hospital of Fudan University (Approval No. 2023-112). Written informed consent was obtained from the legal guardians of all participants prior to sample collection.

### 2.2 Blood sample collection

Peripheral blood was collected into EDTA-containing tubes (BD Vacutainer, USA). Erythrocytes were lysed using red blood cell lysis buffer, and the remaining leukocytes were suspended in TRIzol reagent (Invitrogen, USA) and stored at −80°C for subsequent RNA extraction. For serum collection, whole blood samples were centrifuged at 3000 ×g for 10minutes at 4°C, and the supernatant (serum) was aliquoted and stored at −80°C until use.

### 2.3 Whole-transcriptome sequencing and bioinformatics analysis

#### 2.3.1 Whole-transcriptome sequencing

To clarify the gene expression profile distinguishing KD and FC, we performed RNA-seq on samples from 10 patients in the KD group and 10 age- and sex-matched patients in the FC group using the Illumina NovaSeq 6000 platform. Sequencing was conducted by Sequanta Technologies Co., Ltd. (Shanghai, China), following the manufacturer’s protocols for 2×150 paired-end sequencing. The quality of raw sequencing data was assessed using FastQC (version 0.11.2). Adaptor sequences were trimmed with Trim Galore (version 0.6.7) to generate clean reads. These reads were aligned to the human reference genome (GRCh37, gencode v19 annotation) using STAR (version 2.7.10b). Gene and transcript quantification was performed with RSEM (version 1.2.28) to gather raw counts and fragments per kilobase of exon model per million mapped reads (FPKM) values.

#### 2.3.2 Differential expression analysis and functional enrichment analysis

After that, mRNA-level count data were extracted from the RNA sequencing data using R studio(version 4.3.1), and differential expression analysis was performed using the edgeR package. Differentially expressed genes (DEGs) were identified based on the following criteria: absolute log_2_-fold change (log_2_FC) > 1 and false discovery rate (FDR) < 0.01. Gene Ontology (GO) and Kyoto Encyclopedia of Genes and Genomes (KEGG) enrichment analyses of the DEGs were conducted using the DAVID Bioinformatics website (https://davidbioinformatics.nih.gov/), and bubble plots were generated with the ggplot2 package. Gene Set Enrichment Analysis (GSEA) was performed using the ReactomePA package to identify significantly enriched pathways and extract the leading-edge genes.

#### 2.3.3 Gene set enrichment analysis of ferroptosis-related genes

Based on the classification of ferroptosis-related genes (Drivers, Suppressors, and Markers) provided in FerrDb v2 website (http://www.zhounan.org/ferrdb/current), we applied the GSVA package to assess the relative enrichment of ferroptosis-associated gene sets in the DESs. Specifically, single-sample gene set enrichment analysis (ssGSEA) was performed across all samples. A higher ssGSEA score indicated a higher expression level of the corresponding gene set within a sample, whereas a lower score suggested reduced expression.

#### 2.3.4 Immune cell infiltration analysis of the DEGs

Immune cell infiltration analysis of the DEGs was conducted using CIBERSORTx (https://cibersortx.stanford.edu), and the correlation between ferroptosis leading-edge genes and immune cell proportions was calculated. Following the principle of biological covariate adjustment, immune cells were grouped into three major categories: neutrophils, lymphocytes, and monocyte–macrophages. These immune cell categories were incorporated into the model as covariates to derive ferroptosis scores adjusted for immune cell proportions. The raw sequencing data generated in this study have been deposited in the NCBI Sequence Read Archive (SRA) and are accessible under accession number **PRJNA1338940**.

### 2.4 RNA isolation and qPCR

qPCR was used to validate the expression levels of ferroptosis-related genes in patients with KD and THP-1-Mφ. RNA isolation and qPCR were performed according to the manufacturer’s instructions. The expression levels of DEGs were normalize to the GAPDH level. The primer sequences are provided in **Supplementary Table 1**.

### 2.5 Cell culture and serum stimulation

THP-1 cells were purchased from Shanghai Fuheng Biotechnology Co., Ltd. (Shanghai, China) and maintained in RPMI 1640 medium supplemented with 10% FBS and 1% penicillin–streptomycin (Gibco, USA). The cell line was authenticated by short tandem repeat (STR) profiling. To differentiate THP-1 cells into macrophage-like cells (THP-1-Mφs), the cells were treated with 100 ng/mL PMA (Biosharp, China) for 48 hours. These differentiated cells were then used for subsequent cellular experiments. After differentiation, THP-1-Mφs were stimulated with 20% sera from independent patients from the KD group (KD sera-treated THP-1-Mφs), while sera from HC and FC groups were used as controls.

Liproxstatin-1, a potent ferroptosis inhibitor, primarily acts by suppressing lipid peroxidation (25). Cotreatment with 20 μM Liproxstatin-1 (MCE, USA) was used to evaluate the protective effects of Liproxstatin-1 against KD sera exposure. The results of the concentration exploration of Liproxstatin-1 are shown in **Supplementary Figure 1A**.

### 2.6 Iron assay

The intracellular ferrous iron (Fe^2+^) concentration was measured to assess iron overload, and we compared its changes among different groups. After 24 hours of sera stimulation, the Fe^2+^ concentration in the cells was measured using a Cell Iron Content Assay Kit (Solarbio, China) in accordance with the manufacturer’s instructions. Briefly, cells from each group were collected using extraction buffer and disrupted by ultrasonication. The supernatant was collected, and the protein concentration was determined using a BCA protein assay kit (Takara, Japan). After the reaction buffer was added, the mixture was incubated at 37 °C for 10 minutes, and the absorbance at 593 nm was measured using a microplate reader. The Fe^2+^ concentration was calculated based to the protein concentration.

### 2.7 Lipid peroxidation assay

Lipid peroxidation of the cell membrane is a hallmark of ferroptosis; therefore, we used a BDP 581/591 C11 probe (DOJINDO, Japan) to detect peroxidation in different groups. After 48 hours of sera stimulation, cells were collected and incubated with the BDP 581/591 C11 probe in the dark for 30 minutes at 37 °C. Upon lipid peroxidation, the fluorescence of BDP 581/591 C11 shifted from red (581/610 nm) to green (484/510 nm). The green fluorescence intensity in each group was measured using a flow cytometer and analyzed with FlowJo software.

### 2.8 Mitochondrial membrane potential (MMP, ΔΨm) detection

Mitochondrial membrane potential reflects mitochondrial dysfunction and energy metabolic disruptions, which are downstream effects of lipid peroxidation damage in ferroptosis. Therefore, ΔΨm was measured using a JC-1 assay kit (DOJINDO, Japan) in accordance with the manufacturer’s instructions. After 48 hours of sera stimulation, cells were collected and incubated with 2 μmol/L JC-1 probe in the dark for 30 minutes at 37 °C. Following incubation, the cells were washed three times with HBSS buffer and then resuspended in JC-1 staining buffer. Finally, the percentage of green fluorescence in each group was measured using a flow cytometer and analyzed with FlowJo software.

### 2.9 Western blot analysis

Western blot was used to assess the protein levels of FTH1 in different groups. The detailed procedure for Western blot was descried previously (26). The following antibodies were used: rabbit anti-FTH1 antibody (cat. 3998S; dilution 1:1000; CST, USA), rabbit anti-β-actin antibody (cat. 4970S; dilution 1:1000; CST, USA), and anti-rabbit IgG, HRP-linked antibody (cat. no. 7074S; dilution 1:1,000; CST, USA). β-actin was used as the internal control for each membrane. The raw data are available in **Supplementary Figure 1B**.

### 2.10 Transmission electron microscopy

Transmission electron microscopy (TEM) was performed to assess the morphology of mitochondria associated with ferroptosis in different groups. After 48 hours of sera stimulation and Liproxstatin-1 treatment, THP-1-Mφs in 6-well plates were fixed in 2.5% glutaraldehyde in 0.1 M PBS (pH 7.4) at room temperature. Post-fixation, the cells were incubated with 1% osmium tetroxide prepared in 0.1 M PBS (pH 7.4) for 2 hours at room temperature in the dark. The cells were then dehydrated through a graded series of ethanol, embedded with epoxy resin and sectioned. Ultrathin (80-nm) sections were collected on copper grids and stained with uranyl acetate and lead citrate. Images were acquired using a Hitachi HT7700 transmission election microscope (Hitachi, Ltd.).

### 2.11 Statistical analysis

Statistical analysis was performed using GraphPad Prism (version 9). The choice of statistical test (parametric or nonparametric) was based on the normality of the data and the homogeneity of variance. For qPCR data from patients, Mann–Whitney U test was used, and results are presented as the mean ± standard deviation (SD). For multiple group comparisons, data from cell experiments were analyzed using one-way ANOVA followed by Tukey’s multiple comparisons test and are expressed as the mean ± standard error of the mean (SEM). Paired t tests were used for paired samples.

## 3 Results

### 3.1 Transcriptomic analysis revealed ferroptosis-related molecular features in Kawasaki disease

Baseline clinical characteristics of the patients included in the RNA-seq analysis were summarized in **Table 1** (KD: FC = 10:10). Compared with the FC group, patients with KD exhibited higher platelet counts and C-reactive protein (CRP) levels, but lower hemoglobin and albumin levels. In contrast, some immune-related markers, such as serum amyloid A (SAA), showed no significant differences between the two groups.

**Table 1:** Clinical baseline data of the patients for RNA-seq.

Characteristics	Febrile controls (N = 10)	Kawasaki disease (N = 10)	p
Virus	1/10		
Bacteria	6/10		
Mycoplasma	1/10		
Both virus and Bacteria	1/10		
IKD		3/10	
Sex			
F	5 (50%)	4 (40%)	1.000
M	5 (50%)	6 (60%)	
Age/M	24.0 (23.0, 36.0)	18.0 (11.5, 32.0)	0.121
Temperature	39.2 ± 1.0	39.1 ± 0.6	0.639
HGB	123.7 ± 10.8	98.9 ± 7.3	<0.001
WBC	16.4 (14.5,18.2)	12.9 (10.6,15.5)	0.043
PLT	282.0 (239.0,299.0)	361.0 (330.0,531.0)	0.031
CRP	11.1 (8.0,46.0)	66.8 (39.3,83.0)	0.011
Neu	72.8 ± 9.6	60.2 ± 16.9	0.055
SAA	294.4 (108.4,320.0)	233.9 (105.2,320.0)	1.000
ALB	44.4 ± 3.2	35.9 ± 2.6	<0.001
AST	37.2 (30.8, 44.3)	30.8 (29.7, 33.2)	0.112
ALT	14.0 (13.1, 16.6)	17.1 (12.3, 44.7)	0.853

IKD: Incomplete Kawasaki disease; HGB, Hemoglobin; WBC: White bold cell; PLT, Blood platelet; CRP, C-reactive protein; Neu, Neutrophil; SAA, Serum amyloid A; ALB, Albumin; AST, Aspartate aminotransferase; ALT, Alanine aminotransferase.

**Figure 1:**
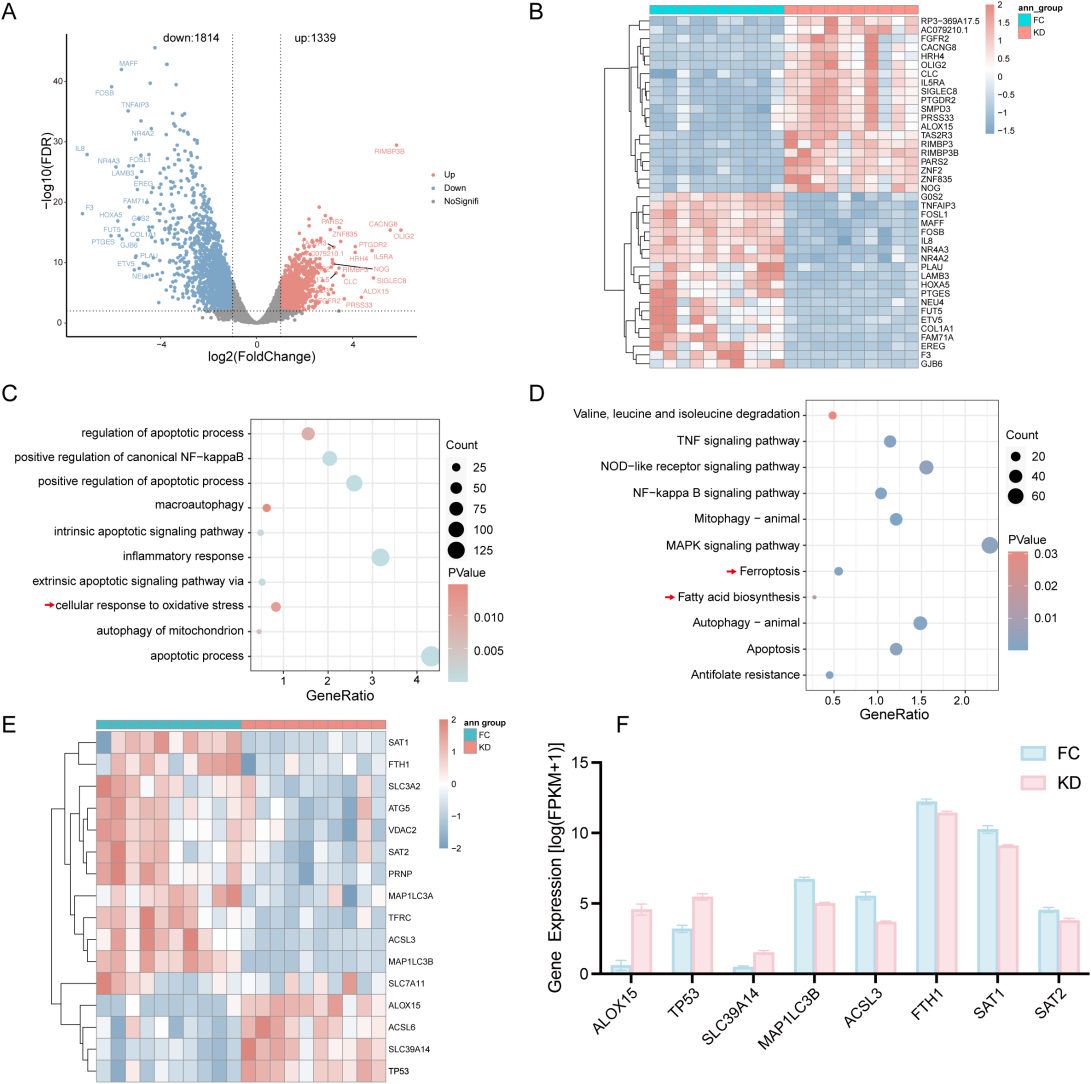
Differential expression analysis of genes between patients with Kawasaki disease and febrile controls. **(A)** Volcano plot showing DEGs. **(B)** Heatmap of the top 20 DEGs; red indicates upregulated genes, and blue indicates downregulated genes. **(C)** GO biological process analysis of all DEGs. **(D)** KEGG analysis of DEGs. **(E)** Heatmap of Ferroptosis-related DEGs between the KD and FC groups. **(F)** Expression levels of Ferroptosis-related DEGs with |log2FC|>1.5 as determined by RNA-seq. KD, patients in acute phase of Kawasaki disease before IVIG treatment; FC, febrile controls; DEGs, Differentially expressed genes.

In the RNA-seq analysis, a total of 3,153 DEGs were identified, including 1,339 upregulated and 1,814 downregulated genes. The distribution of DEGs between the KD and FC groups was shown in **Figure 1A**, and the top 20 upregulated and downregulated genes were displayed in **Figure 1B**.

To explore the biological significance of these DEGs, GO and KEGG enrichment analyses were performed. The DEGs were enriched in inflammation-related pathways, including the NF-κB pathway, inflammatory response, and TNF signaling pathway, as well as in oxidative stress-associated pathways such as cellular response to oxidative stress and mitochondrial autophagy (**Figures 1C, D**). Notably, KEGG analysis revealed that ferroptosis and fatty acid biosynthesis pathways were significantly enriched in the KD group (**Figure 1D**).

According to the results of KEGG, ferroptosis-related genes such as ALOX15, TP53, ACSL6, and SLC39A14 were upregulated in the KD group, whereas genes including SAT1, FTH1, SCL3A2, ATG5, VDAC2, SAT2, PRNP, MAP1LC3A, TFRC, ACSL3, MAP1LC3B and SLC7A11 were downregulated, as shown in the heatmap (**Figure 1E**). A corresponding bar plot further illustrated the expression levels of ferroptosis-related DEGs with |log_2_FC| > 1.5 (**Figure 1F**).

### 3.2 Ferroptosis significantly and specifically activated in Kawasaki disease

**Figure 2:**
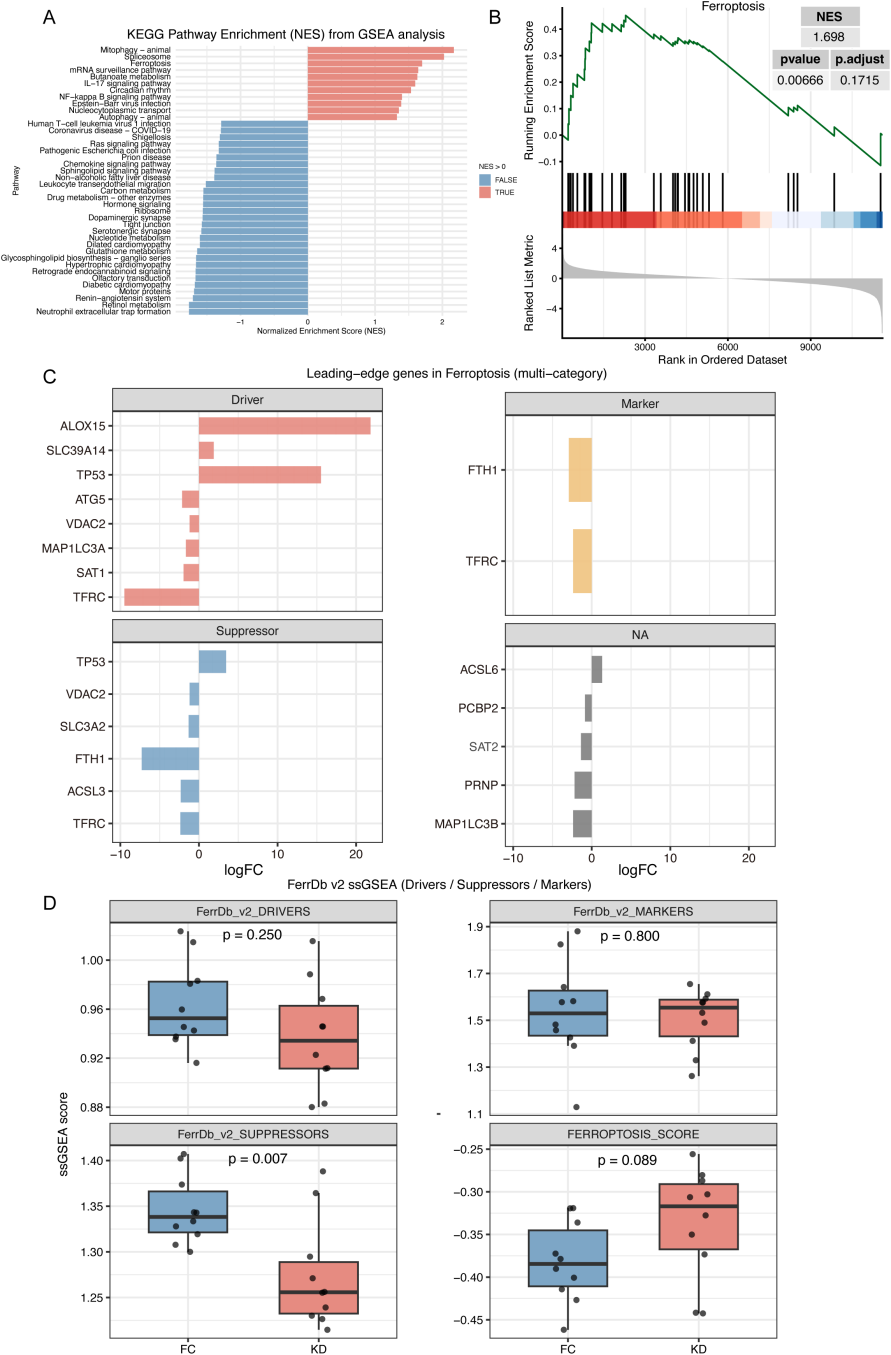
GSEA and ferroptosis score analysis in Kawasaki disease and febrile controls. **(A)** GSEA analysis with KEGG pathway Dataset. This plot shows the enrichment of KEGG pathways with pathways having a positive NES pathway showed in red and those with a negative NES shown in blue. **(B)** GSEA plot demonstrating significant enrichment of the ferroptosis pathway in Kawasaki disease. **(C)** Leading-edge genes of ferroptosis pathway in GSEA analysis. This plot shows the leading-edge genes for ferroptosis based on various categories. **(D)** ssGSEA score for Drivers, Suppressors, and Markers gene sets of ferroptosis. The box plot compares ssGSEA scores for the Driver, Suppressor, and Marker gene sets of ferroptosis between KD and FC groups. The ferroptosis score is calculated as the difference between the Driver score and the Suppressor score. KD, patients in acute phase of Kawasaki disease before IVIG treatment; FC, febrile controls.

GSEA revealed that the ferroptosis pathway was significantly enriched, with a notable NES value (NES = 1.698, p = 0.007, FDR = 0.172), ranking as the third most enriched pathway (**Figures 2A, B**). Among several pathways, including mitophagy, apoptosis, and oxidative phosphorylation, only ferroptosis and mitophagy showed significant enrichment. The leading-edge genes of ferroptosis displayed minimal overlap with those of other pathways, indicating that ferroptosis was independently activated in the KD group (**Supplementary Figure 1C** and **Supplementary Table 2**).

Based on the FerrDb v2 classification of ferroptosis-related genes, activators such as ALOX15 and SLC39A14 were upregulated in the KD group, while suppressors such as SLC3A2, FTH1, and ASCL3 were downregulated. However, some activators, including ATG5, MAP1LC3A, and SAT1, also showed decreased expression. In addition, dual-function genes such as TP53, VDAC2, and TFRC were identified (**Figure 2C**). As showed in **Figure 2D**, ssGSEA analysis further demonstrated an increasing trend in the ferroptosis score in the KD group (Driver-Suppressor, p = 0.089), and a significantly lower score for the Suppressor gene set (Suppressor, p = 0.007), while the Driver gene set scores were comparable between groups (Driver, p = 0.25).

**Figure 3:**
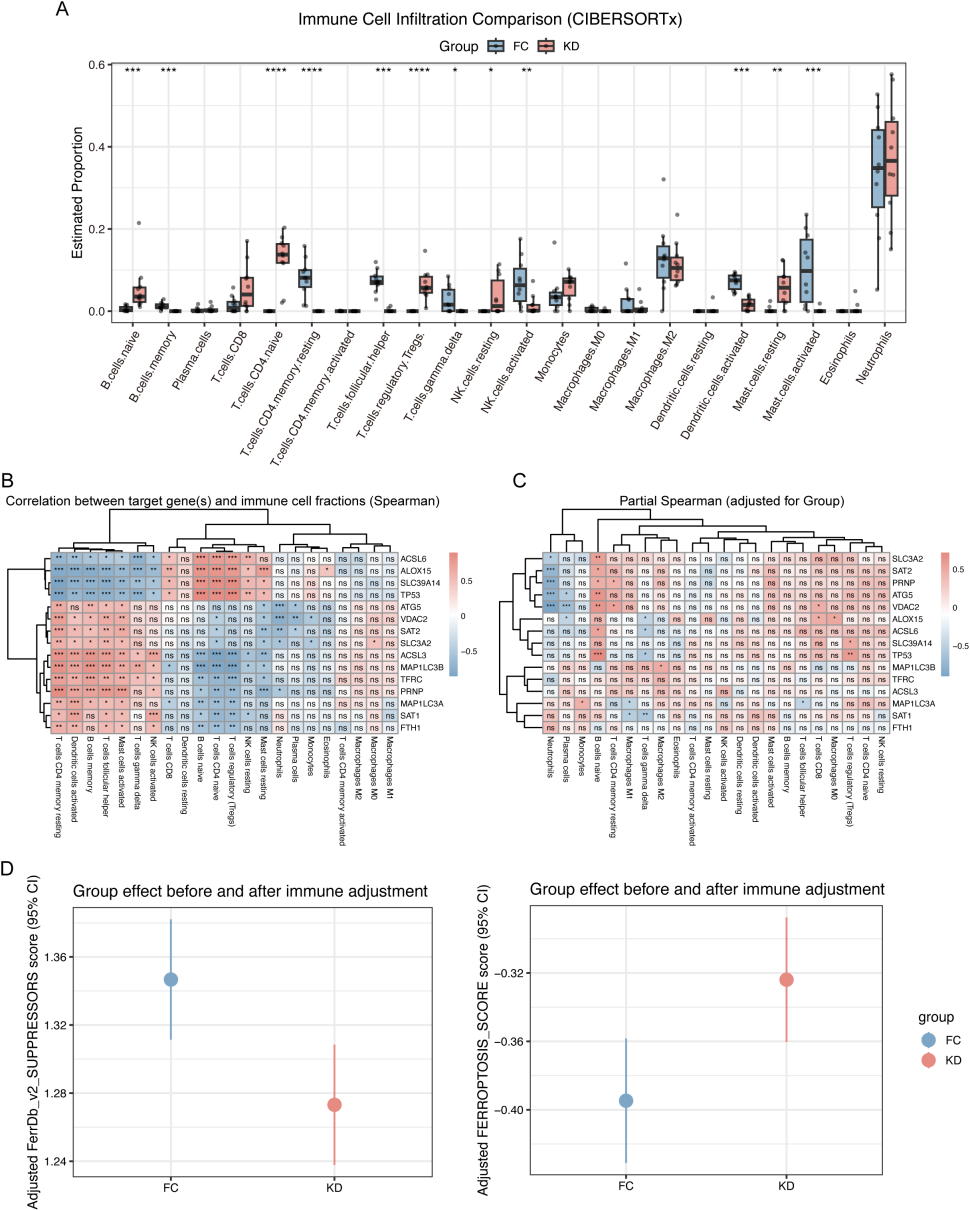
Immune cell infiltration analysis in Kawasaki disease and febrile controls. **(A)** Immune cell infiltration comparison (CIBERSORTx). This boxplot compares the estimated proportions of immune cell types between the KD and FC groups. Significant differences are marked with asterisks, Iindicating the differential immune cell infiltration between the two groups. **(B)** Correlation between leading-edge genes and immune cell fractions. This heatmap shows the Spearman correlation coefficients between the leading-edge genes and immune cell fractions. The correlations are color-coded with blue and red indicating negative and positive correlations, respectively, and “ns“ denotes non-significant correlations. **(C)** Partial Spearman Correlation (Adjusted for Group). This heatmap shows the partial Spearman correlation coefficients between the leading-edge genes and immune cell fractions, adjusted for group effects. The correlations are presented similarly, with blue and red indicating negative and positive associations, respectively. **(D)** Ferroptosis score of group effect after immune cells adjustment. Statistical significance is indicated as follows: ns p > 0.05, *p < 0.05, **p < 0.01, ***p < 0.001, ****p < 0.0001.

Because bulk RNA-seq was performed, potential differences in immune cell composition between the KD and FC groups could have influenced ferroptosis enrichment. Immune cell deconvolution using CIBERSORTx revealed that predominant acute-phase immune cells, such as neutrophils and monocyte–macrophages (27), showed no significant differences between groups. In contrast, lymphocytes, such as naïve B cells, naïve CD4 T cells, and resting NK cells were significantly enriched in the KD group, although these populations are not considered critical effectors during the acute phase (**Figure 3A**). Correlation analysis revealed that ferroptosis leading-edge genes, including ACSL6, ALOX15, SLC39A14, and TP53, were associated with T- and B-lymphocyte subsets (**Figure 3B**). However, after adjusting for group effects, these correlations were largely diminished, particularly for FTH1, ACSL3, and TFRC, suggesting that ferroptosis activation was independent of immune cell composition (**Figure 3C**). Finally, after adjusting ferroptosis and suppressor scores for immune cell proportions, the trends remained consistent with those observed before adjustment (**Figure 2D**), and the 95% confidence intervals of the scores in the KD and FC groups were nearly non-overlapping (**Figure 3D**).

### 3.3 Clinical validation of ferroptosis-related genes expression

**Figure 4:**
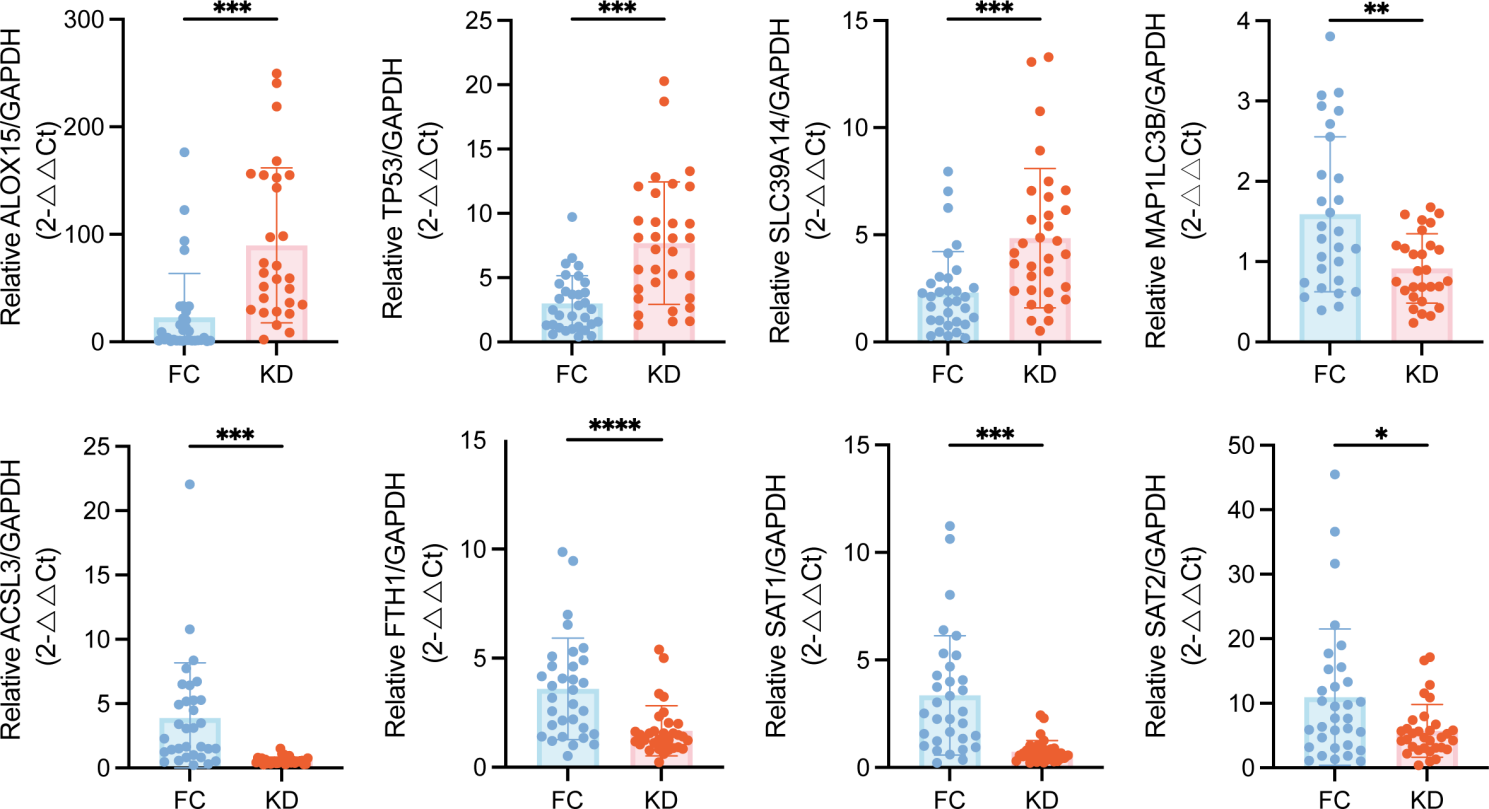
Validation of ferroptosis-related DEGs expression in peripheral blood leukocytes from patients with Kawasaki disease patients and febrile controls. Statistical significance was determined using the Mann–Whitney U test. Statistical significance is indicated as follows: *p < 0.05, **p < 0.01, ***p < 0.001, ****p < 0.0001. n = 32.DEGs, Differentially expressed genes.

To validate these transcriptomic findings, qPCR was performed on selected ferroptosis-related DEGs. As showed in **Figure 4**, the expression levels of *ALOX15*, *TP53*, and *SLC39A14* were elevated in the KD group, consistent with the upregulation observed in the RNA-seq heatmap (**Figure 1E**). In contrast, the expression levels of *MAP1LC3B*, *ACSL3*, *FTH1*, *SAT1* and *SAT2* were decreased, also aligning with the transcriptomic results.

### 3.4 Sera from the KD group induces ferroptosis in THP-1-derived macrophages

**Figure 5:**
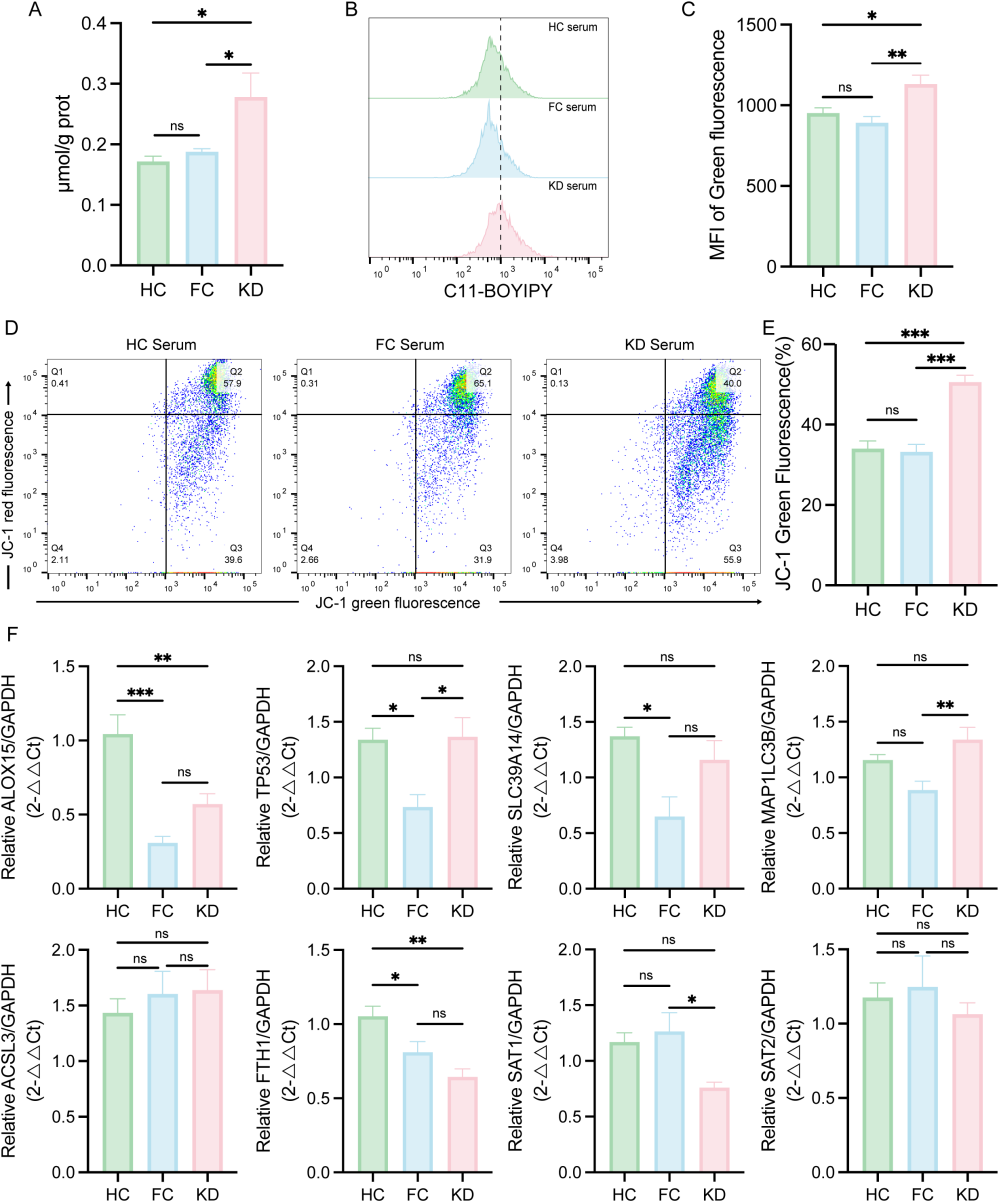
Evaluation of ferroptosis-associated changes in THP-1-Mφs cultured with sera from patients with Kawasaki disease. **(A)** Intracellular Fe^2+^ levels in THP-1-Mφs cultured with different patient sera (n = 5). **(B)** Flow cytometry analysis of lipid ROS in THP-1-Mφs cultured with different patient sera (n = 8). **(C)** Quantification of the mean fluorescence intensity (MFI) for lipid ROS (green fluorescence) analyzed by FlowJo. **(D)** Flow cytometry analysis of the mitochondrial membrane potential (ΔΨm) in THP-1-Mφs cultured with different patient sera (n = 8). **(E)** Percentage of green fluorescent cells (low ΔΨm), as determined by FlowJo. **(F)** Expression of Ferroptosis-related DEGs in THP-1-Mφs cultured with different patient sera (n = 6). The data are presented as the mean ± SEM. Statistical analysis was performed using one-way ANOVA, followed by Tukey’s multiple comparisons test. Statistical significance is indicated as follows: ns p > 0.05, * p < 0.05, ** p < 0.01, *** p < 0.01. KD, sera from patients with Kawasaki disease before IVIG treatment; FC, sera from febrile controls; HC, sera from healthy controls.

Based on the transcriptomic discovery of ferroptosis pathway activation in peripheral blood leukocytes of KD patients, we validated ferroptosis in the *in vitro* KD model by assessing intracellular Fe^2+^ concentration, lipid peroxidation, and ΔΨm. KD sera-treated THP-1-Mφs exhibited significantly increased intracellular Fe^2+^ levels and enhanced lipid ROS levels, compared to cells treated with sera from HC or FC groups (**Figures 5A–C**). In addition, JC-1 staining revealed a marked reduction in ΔΨm in KD sera-treated THP-1-Mφs (**Figures 5D, E**). However, no significant differences in Fe²^+^ levels, lipid ROS, or ΔΨm were observed between the HC and FC groups.

To further explore whether sera from patients with KD modulate the expression of ferroptosis-related DEGs in immune cells, THP-1-Mφs were stimulated with sera from patients in the KD, FC, and HC groups, respectively. As shown in **Figure 5F**, ALOX15, TP53 and SLC39A14 upward trend in expression in the KD group compared with the FC group, but not the HC group. SAT1 and SAT2 also exhibited a decreasing trend in the KD group compared to the FC group, but not the HC group. FTH1 expression was consistently downregulated in the KD group compared to both HC and FC groups. However, other genes such as *MAP1LC3B* and *ACSL3* did not follow the same expression trends as observed in the RNA-seq analysis.

### 3.5 Liproxstatin-1 alleviates KD-induced ferroptosis

**Figure 6:**
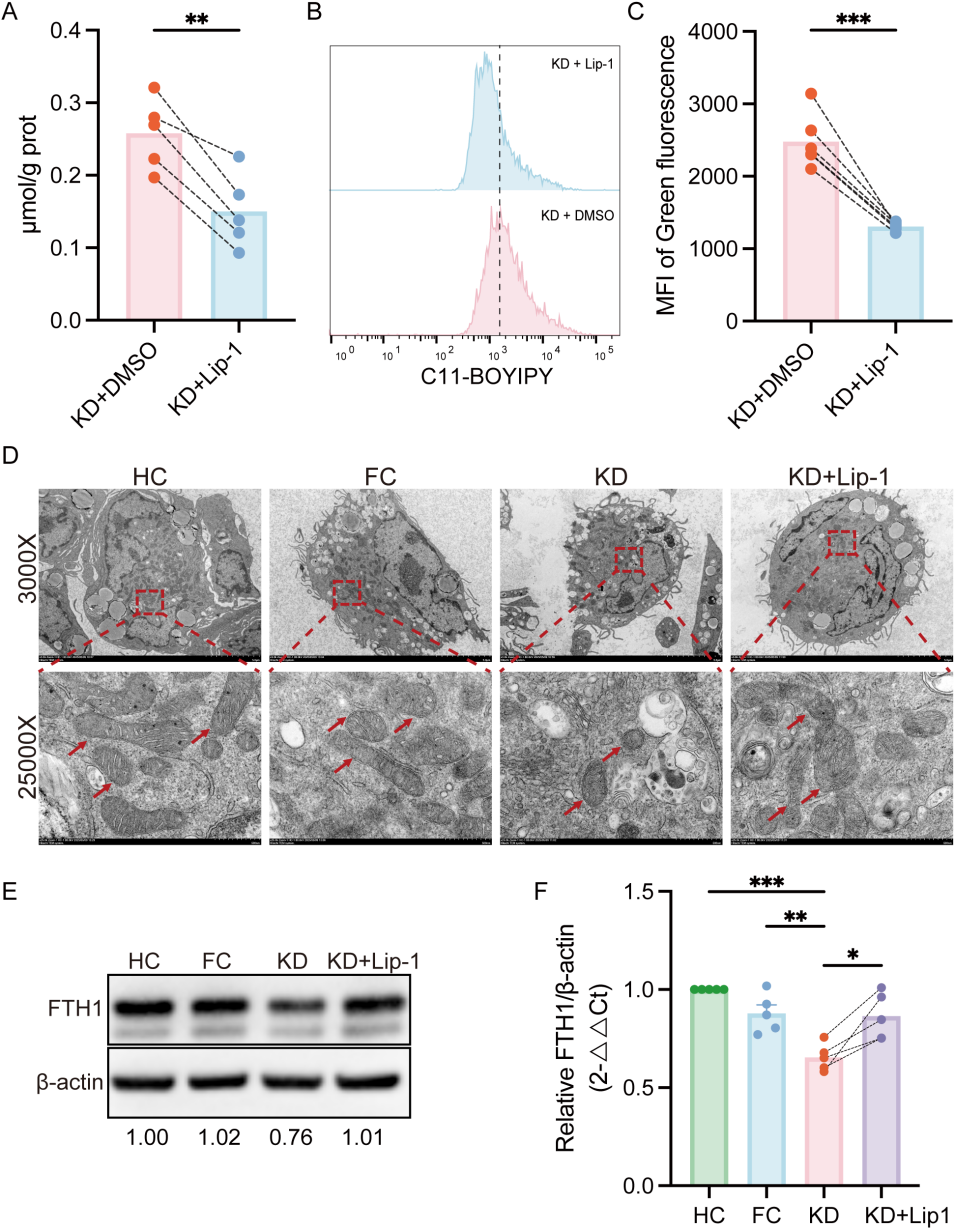
Liproxstatin-1 attenuates ferroptosis induced by KD sera in THP-1-Mφs. **(A)** Intracellular Fe^2+^ levels in THP-1-Mφs treated with Liproxstatin-1 (n = 5). **(B)** Flow cytometry analysis of lipid ROS levels in THP-1-Mφs treated with or without Liproxstatin-1 (n = 6). **(C)** Quantification of the mean fluorescence intensity (MFI) of lipid ROS (green fluorescence), as determined by FlowJo. **(D)** TEM ultrastructural analysis of mitochondrial morphology in THP-1-Mφs. **(E)** WB analysis of protein expression levels of FTH1 in different groups (n = 5). **(F)** Relative quantification of FTH1 protein expression. Statistical analysis: Student’s t test was used for comparisons between the KD and HC/FC groups, and a paired t test was used for comparisons between the KD and KD + Lip-1 groups. Statistical significance is indicated as follows: *p < 0.05, **p < 0.01, ***p < 0.001. Lip-1,Liproxstatin-1; KD, sera from patients with Kawasaki disease before IVIG treatment; KD + Lip-1, KD groups treated with Liproxstatin-1; FC, sera from febrile controls; HC, sera from healthy controls.

To further determine whether ferroptosis contributes to pathological effects induced by KD sera, THP-1-Mφs were co-stimulated with KD sera and Liproxstatin-1, a specific ferroptosis inhibitor. This co-treatment markedly reduced Fe²^+^ accumulation and lipid ROS (**Figures 6A–C**). Subsequently, TEM was used to assess mitochondrial morphology. As shown in **Figure 6D**, mitochondria in THP-1-Mφs treated with KD sera exhibited classical ferroptosis-associated structural changes, including mitochondrial shrinkage, increased membrane density, and loss of cristae, compared to the FC and HC groups. Notably, these ultrastructural abnormalities were partially reversed by Liproxstatin-1 treatment.

FTH1 is a key iron storage protein and negative regulator of ferroptosis. The mRNA-expression of FTH1 was downregulated in patients with KD and also suppressed by KD sera *in vitro*. To validate the involvement of ferroptosis in KD sera-treated THP-1-Mφs, we assessed FTH1 protein levels. As shown in **Figures 6E, F**, FTH1 protein expression was significantly reduced in THP-1-Mφs treated with KD sera compared to those treated with sera from the FC and HC groups. Co-treatment with Liproxstatin-1 partially restored FTH1 protein expression.

## 4 Discussion

In this study, we identified significant alterations in the expression of ferroptosis-related DEGs during the acute phase of KD, providing evidence that ferroptosis may contribute to its pathogenesis. The combined results of ssGSEA and ferroptosis scores suggested that ferroptosis activation in KD may be driven by reduced expression of suppressor/protective genes, playing an important role in the pathogenesis of KD. Sera from patients with KD induced both phenotypic and molecular changes of ferroptosis in THP-1-Mφs, which were partially reversed by the ferroptosis inhibitor Liproxstatin-1. These findings offer novel insights into the underlying mechanism of KD, and suggest ferroptosis as a potential biomarker for diagnosis and a target for therapeutic intervention.

Wang et al. have shown that most DEGs in patients with KD originate from monocytes using single-cell sequencing, including key pro-inflammatory mediators and therapeutic targets such as S100 genes, TNF, and IL1β (28). Therefore, we selected THP-1 cells as the *in vitro* model to explore KD pathogenesis. THP-1 cells have been widely used to study mechanisms related to monocytes and macrophages, as well as immune and inflammatory signaling pathways (29). Compared with peripheral blood mononuclear cells (PBMCs), THP-1 cells more closely resemble human primary monocytes in morphology and function, while also being easier to culture and expand *in vitro*. In addition, they possess a more stable genetic background and avoid the inter-individual variability inherent to PBMCs, thereby enhancing reproducibility of experimental results (29). We attempted to stimulate THP-1 cells with sera from patients with KD to determine whether ferroptosis could be induced. Consistent with the transcriptomic analysis, mRNA levels of FTH1 were downregulated compared to HC and FC groups. However, some genes did not fully align with the transcriptome results, which may be attributable to intrinsic gene expression differences between THP-1 cells and peripheral leukocytes from patients with KD. Nevertheless, these discrepancies do not undermine the evidence supporting ferroptosis activation in KD. Since monocytes mature into macrophages and play an important role in immune responses, THP-1 cells were differentiated into adherent macrophages (THP-1-Mφ) using PMA for subsequent experiments.

Oxidative stress is a well-recognized contributor to the pathophysiology of KD. Uchida et al. reported elevated plasma lipid peroxide levels during the acute phase of KD, along with increased manganese superoxide dismutase activity in lymphocytes and increased glutathione peroxidase and catalase activity in erythrocytes (30). Recent studies have further elucidated its mechanistic role: Jin et al. demonstrated that neutrophil extracellular traps activate the NLRP3 inflammasome and induce pyroptosis via ROS-dependent pathways (31), while Yao et al. reported that silencing *NFATC1* alleviates oxidative stress in human coronary artery endothelial cells exposed to sera from KD patients (32). Excessive or dysregulated intracellular iron generates reactive ROS via Fenton reactions, leading to lipid peroxidation and cellular injury (33, 34). Impaired antioxidant defenses, such as reduced glutathione peroxidase 4 (GPX4) activity, further promote ferroptosis by limiting lipid peroxide clearance (33, 35). This creates a feed-forward loop in which oxidative stress and ferroptosis exacerbate each other (36). Consistent with this interplay, previous reports of elevated serum ferritin in patients with KD support the involvement of iron metabolism in disease pathology. Our RNA-seq analysis aligns with these observations, revealing significant enrichment of ferroptosis-related pathways in the KD group compared to the FC group. Furthermore, by examining the overlap of leading-edge genes among GSEA-enriched pathways, we found that ferroptosis represented an activation pathway largely independent of oxidative stress.

In our study, THP-1-Mφs exposed to KD sera exhibited increased intracellular Fe²^+^ concentrations and elevated levels of lipid ROS, two central features of ferroptosis. These changes were not observed in cells treated with HC or FC sera, suggesting a specific effect of KD sera. As mitochondrial dysfunction plays a critical role in ferroptosis-induced lipid peroxidation (37–39), we examined mitochondrial ultrastructure by TEM and observed typical ferroptosis-associated mitochondrial alterations induced by KD sera. Importantly, these phenotypes were alleviated by Liproxstatin-1, further supporting the role of ferroptosis in the cellular response to KD sera. These findings also suggest that the observed increase in Fe²^+^ and lipid ROS levels likely result from ferroptosis rather than other forms of cell death.

At the molecular level, ferroptosis is regulated by a network of genes involving in lipid metabolism, iron homeostasis, redox balance, and antioxidant defenses (10). *FTH1* encodes the heavy chain of ferritin, which catalyzes the oxidation of Fe^2+^ to Fe^3+^. Reduced FTH1 expression suggests diminished iron storage capacity and increased intracellular Fe^2+^ levels (40–42), which is considered a characteristic molecular feature of ferroptosis. In patients with KD, both *FTH1* mRNA and protein levels were markedly decreased, and Liproxstatin-1 treatment partially restored its expression, suggesting a potential protective role through *FTH1*-related pathways. *ALOX15* encodes 15-lipoxygenase, an enzyme that promotes lipid peroxidation and drives ferroptosis (43, 44). Its upregulation in KD indicates enhanced lipid peroxidation and increased susceptibility to ferroptosis. *SLC7A11* encodes a subunit of the cystine/glutamate antiporter, which supports glutathione synthesis and suppresses ferroptosis, whereas TP53, a tumor suppressor protein, promotes ferroptosis by repressing SLC7A11 expression (33, 45, 46). In our dataset, *TP53* upregulation combined with *SLC7A11* downregulation indicates *TP53*-mediated enhancement of ferroptosis. Although certain pro-ferroptosis genes, such as *SAT1* (43), were downregulated, the overall gene expression profile in KD favors ferroptosis. To determine whether ferroptosis pathway enrichment was influenced by immune cell composition, we adjusted ferroptosis enrichment scores for immune cell proportions. The results showed that significant enrichment of ferroptosis pathway was independent of immune cell composition, further confirming the involvement of ferroptosis in KD. Based on the FerrDb v2 classification of ferroptosis-related genes, ssGSEA analysis demonstrated a significantly lower score for the Suppressor gene set in the KD group, whereas the ferroptosis score (calculated as the difference between Driver and Suppressor scores) was elevated. This suggests that reduced expression of ferroptosis-protective genes may play a more critical role in KD pathogenesis. Overall, this pattern is characterized by upregulated pro-ferroptosis genes and downregulated protective genes, likely reducing antioxidant capacity and increasing ferroptosis susceptibility. Further studies are warranted to elucidate the specific contributions of these genes to KD pathogenesis.

KD often presents with non-specific febrile symptoms in the acute phase, making it difficult to differentiate from other febrile illnesses (47). Comparative analysis between KD and other febrile illnesses may enhance our understanding of KD-specific mechanisms. A previous study compared ferroptosis-related gene expression profile in KD and healthy controls, identifying genes such as *MAPK14*, *SLC2A3*, and *PGD* as potential diagnostic biomarkers (48). In contrast, our study used febrile illness controls to identify DEGs that distinguish KD from other febrile illnesses. Notably, *MAPK14*, *SLC2A3*, or *PGD* did not differ significantly between KD and FC groups in our dataset, suggesting these genes reflect generalized febrile inflammation rather than KD-specific processes.

This study has several limitations. First, the experiments were conducted exclusively in cellular models. The primary objective of this study, however, was to explore whether ferroptosis activation occurs in KD. Future studies should incorporate animal models to validate our findings, elucidate the specific molecular mechanisms of ferroptosis in KD, and identify potential therapeutic targets. Second, our investigation focused exclusively on the acute phase of KD; it remains unclear whether ferroptosis contributes to the progression or resolution of CALs. Third, although our results implicate the involvement of ferroptosis in KD, other forms of cell death, such as pyroptosis, apoptosis, and necroptosis, cannot be excluded from the pathophysiology of KD and should be further investigated.

## 5 Conclusion

With RNA-seq analysis and *in vitro* experiments, this study revealed significant enrichment of ferroptosis-related genes and activation of ferroptosis-associated pathways in KD. Sera from patients with KD promoted ferroptosis in macrophages, as evidenced by elevated intracellular iron levels, increased lipid peroxidation, and mitochondrial dysfunction. Notably, treatment with Liproxstatin-1 alleviated these changes by partially restoring *FTH1* expression, and improving mitochondrial morphology, suggesting ferroptosis inhibition as a potential therapeutic approach for KD.

## Data Availability

The datasets presented in this study can be found in online repositories. The names of the repository/s and accession number(s) can be found below: PRJNA1338940 (SRA).
