# Supplementary material for: Ferroptosis-related oxidative stress activation in the acute phase of Kawasaki disease
Source: Front Immunol. 2025 Nov 13;16:1704978. doi: 10.3389/fimmu.2025.1704978 (PMC12657192; doi:10.3389/fimmu.2025.1704978)
Supplement: Supplementary file 1 [file Table1.docx]

Supplementary Material

# Supplementary Figures and Tables

## Supplementary Table

Supplementary Table 1 Primer Sequences

| Gene | Forward Primer | Reverse Primer |
| --- | --- | --- |
| GAPDH | GGAGCGAGATCCCTCCAAAAT | GGCTGTTGTCATACTTCTCATGG |
| ALOX15 | GGGGCAAGGAGACAGAACTC | GCGGTAACAAGGGAACCTGA |
| TP53 | ACCTATGGAAACTACTTCCTGAAA | CTGGCATTCTGGGAGCTTCA |
| SLC39A14 | TCCGAGCGCCAGGTTTATTC | CCATAAGCCAAGCAGGGTCA |
| MAP1LC3B | TTCAGGTTCACAAAACCCGC | TCTCACACAGCCCGTTTACC |
| ACSL3 | TGACACAAGGGCGCATATCT | CCAGTCCTTCCCAACAACGA |
| FTH1 | CCAGAACTACCACCAGGACTC | GAAGATTCGGCCACCTCGTT |
| SAT1 | TGGTCCGCAAAGGGAAGAAA | ATCAGCCGCAGTATGTCACT |
| SAT2 | GGCTGATTCGGGTGAAGACT | CCCTTGACCCCGATATTCCG |

| ID | Description | NES | p-value | p.adjust | Leading-edge genes |
| --- | --- | --- | --- | --- | --- |
| hsa04137 | Mitophagy - animal | 2.171 | <0.001 | <0.001 | HIF1A/TOMM40L/GABARAPL1/SIAH1/JUN/RELA/MAP1LC3B/USP30/RAB5A/UBC/ATF4/TOMM20/EIF2AK3/TBC1D15/SMURF1/FOXO3/NRAS/TAX1BP1/CALCOCO2/ARIH1/RABGEF1/MON1A/TP53/TBK1/E2F1/USP8/MAP1LC3A/MUL1/VCP/RAB5C/OPA1 |
| hsa04216 | Ferroptosis | 1.698 | 0.007 | 0.172 | MAP1LC3B/PRNP/TFRC/ACSL3/SAT1/VDAC2/SAT2/TP53/MAP1LC3A/ATG5/SLC3A2/SLC39A14/FTH1/ALOX15/PCBP2/ACSL6 |
| hsa04215 | Apoptosis-multiple species | -0.724 | 0.844 | 0.925 | BCL2L11/NGFR/BCL2L1/MAPK8/MAPK9/BAX/XIAP |
| hsa04210 | Apoptosis | -0.972 | 0.506 | 0.726 | GZMB/BCL2L1/NOL3/PIK3R2/IL3RA/TUBA1B/MAPK8/CTSO/PIK3CD/CAPN2/PDPK1/MAP3K5/CSF2RB/MAPK9/MAPK3/MAP2K2/AKT1/IKBKG/PARP3/TUBA8/TNFRSF10B/CASP10/BAX/CFLAR/PARP2/XIAP/CTSD/ACTB |
| hsa00190 | Oxidative phosphorylation | -1.040 | 0.391 | 0.620 | NDUFA13/NDUFB11/NDUFA12/PPA2/NDUFB8/NDUFS2/NDUFC2/COX7C/NDUFB7/NDUFAB1/COX5A/UQCRC1/NDUFC1/COX7A2L/ATP6V1B2/ATP6V0E1/NDUFA11/UQCRQ/COX6B1/NDUFS4/SDHD/ATP6V1E1/NDUFB3/NDUFB10/NDUFB6/UQCR10 |
| hsa05208 | Chemical carcinogenesis-reactive oxygen species | -1.227 | 0.098 | 0.356 | PIK3R2/NDUFC2/MAPK8/COX7C/GSTM3/GSTO1/ABL1/AKR1C3/MAPK11/NDUFB7/NDUFAB1/COX5A/VDAC1/PIK3CD/PDPK1/UQCRC1/MAP3K5/NDUFC1/PRKCD/COX7A2L/MAPK9/MAPK3/GSTT1/GSTO2/MAP2K2/AKT1/NDUFA11/MAPK13/UQCRQ/AKR1A1/COX6B1/ABL2/IKBKG/NDUFS4/MGST1/SDHD/NDUFB3/NDUFB10/MAPK12/NDUFB6/UQCR10/PTK2/MAPK14 |
| hsa04217 | Necroptosis | -1.329 | 0.070 | 0.307 | IFNAR1/PYCARD/MLKL/TYK2/CHMP3/PYGM/CHMP7/CHMP5/MAPK8/PPIA/TLR4/CAMK2D/CYBB/VDAC1/CAPN2/SMPD1/PYGL/RIPK3/CHMP4A/MAPK9/JAK2/SHARPIN/JAK3/IL1B/RNF31/CASP1/TNFRSF10B/BAX/CFLAR/TICAM2/JAK1/XIAP/PLA2G4B/STAT5B |

**Supplementary Table 2 GSEA Results for Ferroptosis and Other KEGG Pathways in KD**

## Supplementary Figures


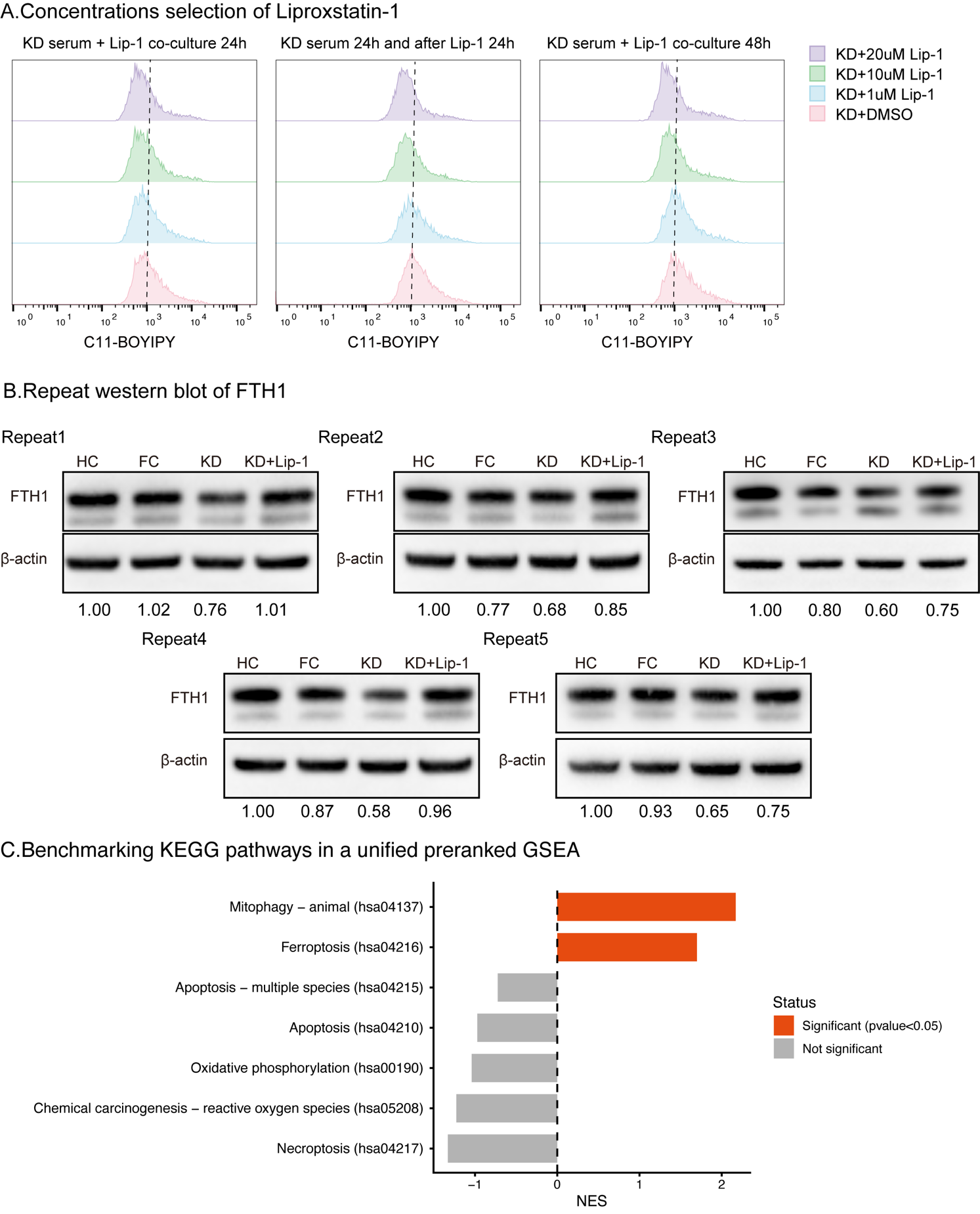


**Supplementary Figure 1.** A. Concentrations selection of Liproxstatin-1. THP1-Mφ cells were treated under different conditions: (i) co-stimulation with KD sera and Lip-1 for 24 h, (ii) KD sera stimulation for 24 h followed by Lip-1 treatment for another 24 h, and (iii) co-stimulation with KD sera and Lip-1 for 48 h. Lip-1 was applied at concentrations of 0 μM, 1 μM, 10 μM, and 20 μM. Lipid ROS levels were subsequently measured, and 20 μM was identified as the optimal therapeutic concentration. B. Representative results of FTH1 protein from five independent biological replicates. C. Benchmarking of KEGG pathways in a unified pre-ranked GSEA.
